# Cytocompatibility of Polymers for Skin-Contact Applications Produced via Pellet Extrusion

**DOI:** 10.3390/jfb15070179

**Published:** 2024-06-29

**Authors:** Sakine Deniz Varsavas, Paweł Michalec, Mohammed Khalifa, Ping Li, Sebastian Spintzyk

**Affiliations:** 1 ADMiRE Research Center, Carinthia University of Applied Sciences, Europastraße 4, 9524 Villach, Austriap.michalec@fh-kaernten.at (P.M.); s.spintzyk@fh-kaernten.at (S.S.); 2 Competence Center for Wood Composites and Wood Chemistry, Wood K Plus, Klagenfurter Str. 87–89, 9300 St. Veit an der Glan, Austria; m.khalifa@wood-kplus.at; 3 Department of Prosthodontics, School and Hospital of Stomatology, Guangdong Engineering Research Center of Oral Restoration and Reconstruction, Guangzhou Medical University, Guangzhou 510180, China; 4 Guangzhou Key Laboratory of Basic and Applied Research of Oral Regenerative Medicine, Guangzhou Medical University, Guangzhou 510180, China

**Keywords:** additive manufacturing, orthoses and prostheses, pellet extrusion, biocompatibility, skin-contact application

## Abstract

Orthoses and prostheses (O&P) play crucial roles in assisting individuals with limb deformities or amputations. Proper material selection for these devices is imperative to ensure mechanical robustness and biocompatibility. While traditional manufacturing methods have limitations in terms of customization and reproducibility, additive manufacturing, particularly pellet extrusion (PEX), offers promising advancements. In applications involving direct contact with the skin, it is essential for materials to meet safety standards to prevent skin irritation. Hence, this study investigates the biocompatibility of different thermoplastic polymers intended for skin-contact applications manufactured through PEX. Surface morphology analysis revealed distinct characteristics among materials, with TPE-70ShA exhibiting notable irregularities. Cytotoxicity assessments using L929 fibroblasts indicated non-toxic responses for most materials, except for TPE-70ShA, highlighting the importance of material composition in biocompatibility. Our findings underscore the significance of adhering to safety standards in material selection and manufacturing processes for medical devices. While this study provides valuable insights, further research is warranted to investigate the specific effects of individual ingredients and explore additional parameters influencing material biocompatibility. Overall, healthcare practitioners must prioritize patient safety by meticulously selecting materials and adhering to regulatory standards in O&P manufacturing.

## 1. Introduction

Orthoses and prostheses (O&P) are medical assistive devices supporting people in need. Orthoses are devices that aim to treat deformities by supporting the joints or improving the functionality of the limb, while prostheses are artificial limbs that replace a missing body part. Orthotic devices can be classified into three main categories, namely, the spinal orthosis, upper limb orthosis (shoulder, arm, elbow, wrist, and hand orthoses), and lower limb orthosis (foot, ankle–foot, knee, knee–ankle–foot, and hip–knee–ankle–foot orthoses) [1]. On the other hand, prostheses are classified depending on where the amputation is performed: upper extremity (trans-carpal, wrist disarticulation, trans-radial elbow disarticulation, transhumeral, shoulder disarticulation, and forequarter) [2] and lower extremity (hemipelvectomy, hip disarticulation, above-knee, below-knee, and symes) prostheses [3]. Independent of the level of deformity or amputation, proper selection of material for orthotic and prosthetic devices is essential. For instance, the materials used to produce O&P should have specific mechanical properties to withstand daily use, be light, and, most importantly, be biocompatible [4].

Recent advancements in the field of biomaterials have enabled the fine-tuning and tailoring of both the physical and biological properties of materials. These innovations demonstrate the significance of comprehensive evaluations that encompass mechanical properties as well as biocompatibility [5]. Moreover, according to ISO 22523 [6], the materials that will be used for external limb prostheses and orthoses should not cause any irritation or sensitization; furthermore, any material in contact with the human body should be assessed in terms of biocompatibility. According to the biocompatibility point of view, biomaterials can be categorized as biologically inert materials (first generation, i.e., mostly used in O&P applications), bioactive and biodegradable materials (second generation, i.e., showing biological reactions with bones to make bonds), and bioresorbable and bioactive materials (third generation, i.e., stimulating tissue regeneration by tuning into the material composition) [7]. Materials used for O&P applications can be classified as first-generation biomaterials. The most commonly used materials in the manufacturing of O&P are metals; leather; wood; thermoplastics, e.g., polyetheretherketone (PEEK), polyethylene (PE), and polypropylene (PP); thermoplastic elastomers (TPE); thermosets, e.g., polyurethane (PU), epoxy resins, polyester resins, and their fiber reinforced composites, e.g., carbon, glass, aramid, and natural fibers [4,8]. Conventional O&P manufacturing is based on the thermoforming of polymer sheets and the hand lay-up of fiber-reinforced pre-pregs on a plaster-casted base [4,9,10,11]. However, the patient’s acceptance of the O&P, which are manufactured with traditional methods, strongly depends on the technician’s expertise, and it is a time-consuming process. Therefore, in case of a need for modifications or renewal, reproducibility is impossible because no digital data are available to replicate the O&P [9].

According to a recent market analysis by Research and Markets [12], the share of the O&P market is expected to be USD 9 billion by 2028 due to the increased number of road accidents, sports injuries, and amputations due to diabetes-related diseases. Moreover, in the same analysis, it was reported that the rising technological advancements in additive manufacturing (AM) are likely to promote the growth of the O&P market because of the ease of production and lower costs. AM technology offers a cost-effective and simplified workflow for creating customized parts with the possibility of using many materials [13]. With this technology, it is possible to combine soft, rigid, and smart materials, which have some additional features such as shape memory or electrical conductivity [9,13,14,15,16,17,18]. In addition to the many types of AM technologies, the pellet extrusion (PEX) method for skin contact applications offers many possibilities (as there is a large selection of materials), which include the printing of very soft materials with hardnesses of 30 Shore00 and 45 ShoreA (which is not possible with filament extrusion processes) [19]. Furthermore, multi-material AM allows the combination of stiffer materials with soft ones to achieve a broader range of properties [20]. However, to the best of our knowledge, the biocompatibility of the materials after extrusion with PEX printing has not been investigated in the literature. The expanded selection of materials for medical purposes offers advantages in terms of design flexibility and device property optimization.

The aim of this study was to investigate the possibility of using different materials for skin-contact applications produced with the PEX method. Specifically, it aimed to determine whether the PEX method can influence the biocompatibility of these materials, beyond their inherent properties, due to potential factors like alterations of surface morphology, potential reductions in residual stresses, or the introduction of specific surface characteristics that might improve cell attachment and proliferation. This involved testing and verifying the cytocompatibility of several materials with a wide range of hardnesses. Furthermore, the surface morphologies of specimens were observed using scanning electron microscopy, and surface roughness was analyzed. Cytotoxicity evaluations were performed using both direct contact tests and extract tests to comprehensively assess the influence of the PEX method on the material suitability of O&P for skin-contact applications.

## 2. Materials and Methods

### 2.1. Specimen Preparation

In this study, seven different materials from three suppliers were tested: Pollen AM (Ivry sur Seine, France), Kraiburg TPE (Waldkraiburg, Germany), and HEXPOL (Malmö, Sweden). All the materials are listed in Table 1. According to the technical data sheets, selected materials are polymers containing additives that are included to enhance properties such as flexibility, strength, and durability, which are crucial for O&P applications. Moreover, they are intended for general applications ranging from general consumer products such as food serviceware, toys, infant care products, food containers, packaging, and sports equipment to specific industrial applications such as seals, grommets, belts, and electric and electronic applications. Furthermore, one of the materials, i.e., TPE-EC, is electrically conductive thermoplastic elastomeric material that can be used for sensorized O&P production via PEX-based AM. In the selection of materials, general-grade materials were considered to explore their potential of customized O&P applications after processing with PEX-based AM. Materials used in conventional O&P manufacturing provide a broad basis; therefore, evaluation of the versatility and suitability of alternative materials for skin-contact applications is essential for ensuring comfort, functionality, and safety in O&P devices that come into direct contact with the skin. 

**Figure 1:**
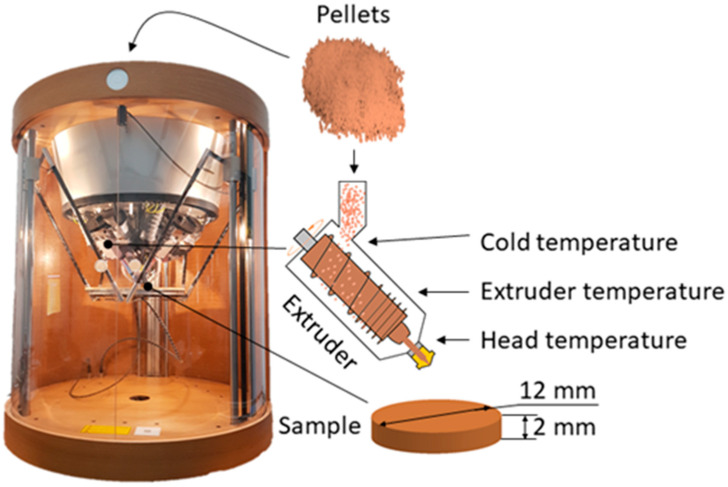
Pellet 3D printer with indicated heating zones.

The cylinderical test specimens were designed with the following dimensions: 12 mm diameter and 2 mm thick. All test specimens were produced with the pellet 3D printer Pollen New PAM Series P (Pollen AM, Ivry sur Seine, France). This printer has three heating zones on the extruder screw to ensure better pellet extrusion (Figure 1). The printing parameters for all the materials are summarized in Table 2. All the specimens were printed with 0.4 mm nozzle size, 0.2 mm layer height, 0.4 mm line width and 100% zigzag infill. There was no retraction or fan cooling set.

### 2.2. Surface Characterization 

Surface morphologies were observed using scanning electron microscopy (SEM). Prior to SEM, specimen surfaces were sputter-coated with a thin layer of gold–palladium. The surface morphologies of the printed samples were observed using a scanning electron microscope (SEM, TESCAN, Brno, Czech Republic) at an acceleration voltage of 15 kV.

Addional roughness data were evaluated using a confocal 3D laser scanning microscope (Keyence VK-X1000, Osaka, Japan; software: VK-H1XME V2.2.) with coaxial lighting at various magnifications (lens: 20× lens; z-axis step size: 0.75 µm; image field: 10 × 11 images (approx. 5075 × 6145 µm)). Surface roughness was measured by considering at least 10 different zones. Each zone was measured three times, and the average of the three measured values was considered as the surface roughness for that zone.

### 2.3. Cytotoxicity Test

Mouse fibroblasts (L929, Procell Life Science and Technology, Wuhan, China) were used for the cytotoxicity test. L929 fibroblasts were cultured in Dulbecco’s Modified Eagle Medium (DMEM, Gibco, Grand Island, NY, USA) with 10% fetal bovine serum (FBS, Gibco, Grand Island, NY, USA) and penicillin and streptomycin (100 U/mL) (PS, Gibco, Grand Island, NY, USA). L929 cells were grown under standard cell culture conditions (37 °C, 5% CO_2_, and 95% relative humidity). The cytotoxicity test was performed via the extract test and direct contact test, according to ISO 10993-5: 2009 [21] and ISO 10993-12: 2012 [22].

The complete cell culture medium was used for the extraction medium. The ratio of surface area to the extraction medium was set to 1.25 cm^2^/mL. Tested specimens were immersed for 72 h under cell culture conditions. Titanium (Ti)-based alloy and pure copper (Cu) discs were used as the negative and positive controls, respectively. Afterward, extracts were disinfected using a filter (0.2 μm).

The live/dead fluorescence staining was performed to qualitatively investigate cell morphology. L929 fibroblasts were seeded in a 24-well plate at a cell density of 3 × 10^4^ cells/cm^2^ and cultured overnight. Next, the medium was replaced with the respective extracts. After incubation for 24 h, samples were rinsed with phosphate-buffered saline (PBS, Gibco, Grand Island, NY, USA). Cells were fluorescently stained using 1 mL of staining reagent containing 1 μM calcein acetoxymethyl (Calcein AM) and 4 μM propidium iodide (PI) for 10 min in darkness. Cells were observed using a fluorescence microscopy (DMi8, Leica Microsystems GmbH, Wetzlar, Germany).

The quantitative assessment was determined using relative cell metabolic activity and lactate dehydrogenase (LDH) release. Specifically, fibroblasts were seeded into a 96-well plate at a density of 3 × 10^4^ cells/cm^2^. After 24 h, the medium was replaced with 100 μL sample extracts. After incubation for another 24 h, a cell counting kit-8 assay (CCK-8, Dojindo Laboratories CO., Kumamoto, Japan) and an LDH assay (Beyotime Biotechnology, Nanjing, China) were used according to the manufacturers’ instructions. The relative metabolic activity and relative LDH release were calculated, as previously reported in detail [23,24].

Prior to the direct contact test, specimens were ultrasonically cleaned and disinfected in 70% ethanol for 10 min. Ti and Cu discs were used as the negative control and positive control. Briefly, three specimens per group were processed in parallel using 24-well culture plates. L929 cells were seeded at a density of 2 × 10^4^ cells/cm^2^. The cell viability was determined after incubation for 24 h. Cell morphology, cell metabolic activity, and relative LDH release were determined, as described above. All biological tests were independently repeated in triplicate.

### 2.4. Statistical Analysis

Descriptive data were shown as the mean and standard deviation. One-way analysis of variance (ANOVA) followed by Tukey’s post hoc multiple comparisons test was used to determine statistically significant differences. GraphPad PRISM version 6.1 (GraphPad Software, Inc., San Diego, CA, USA) was used for statistical analyses. Statistical significance was set to a significant level of α = 0.05.

## 3. Results

### 3.1. Surface Morphologies

**Figure 2:**
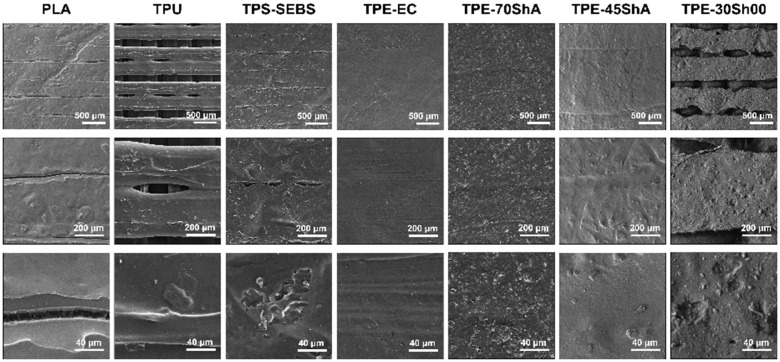
Representative SEM images of additively manufactured specimens (magnification 30×, 100× and 500×).

Figure 2 presents the representative SEM images of different specimen surfaces at different magnifications. Regarding the specimens of TPU and TPE-30Sh00, regular texture layer-by-layer surfaces and regular gaps in between could be observed. Furthermore, the layers underneath the top layers are clearly visible. In the PLA group, the individual layers were still clearly visible. However, the gaps between them were much narrower than in the TPU group. Those smaller gaps were not contiguous in TPS-SEBS, and the printed layers seemed more connected to each other. In comparison, the remaining groups (i.e., TPE-EC, TPE-70ShA, and TPE-45ShA) exhibited relatively smooth surfaces without apparent fissures and a more homogeneous surface. The top layers show fully connected layers (closed surfaces).

**Figure 3:**
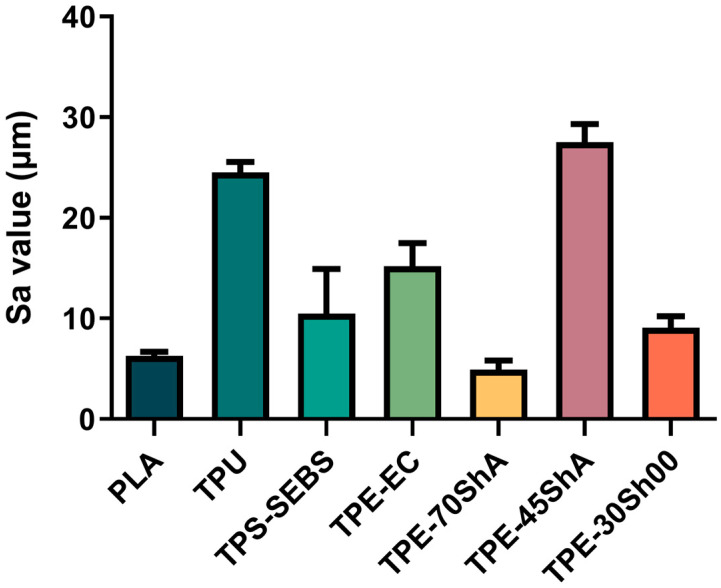
Representative average roughness of all groups.

Figure 3 shows the representative average roughness (Sa) of tesed specimens. The lowest values were determined for the TPE-70ShA (4.93 µm) and PLA (6.28 µm) groups. In the TPS-SEBS, TPE-EC, and TPE-30Sh00 groups, the Sa values were between 10 µm and 20 µm. In addition, the highest values were measured for the TPU (24.52 µm) and TPE-45ShA (27.50 µm) groups.

### 3.2. Cytotoxicity Evaluation

**Figure 4:**
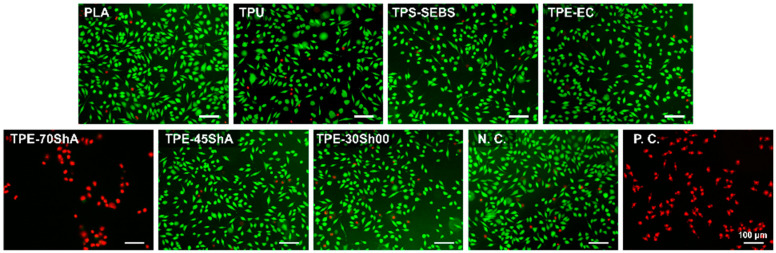
Representative fluorescent staining images of cells cultured in specimen extracts for 24 h (scale bar = 100 μm). Ti and Cu were the negative control (N.C.) and positive control (P.C.), respectively. Green fluorescence represents viable cells, and red fluorescence indicates apoptotic cells with compromised membrane integrity.

To determine the potential cytotoxic effects, L929 fibroblasts cultured in extracts were evaluated using live/dead staining, as illustrated in Figure 4. Concerning the extract of the TPE-70ShA group, most stained cells showed red fluorescence, similar to the positive control, indicating cell apoptosis with compromised membrane integrity. In addition, concerning the other extracts of the 3D-printed specimens, green-stained fibroblasts exhibited a spindle shape with clear cellular outlines, which had no apparent differences compared with the negative control. This indicated that these extracts had no apparent cytotoxicity.

**Figure 5:**
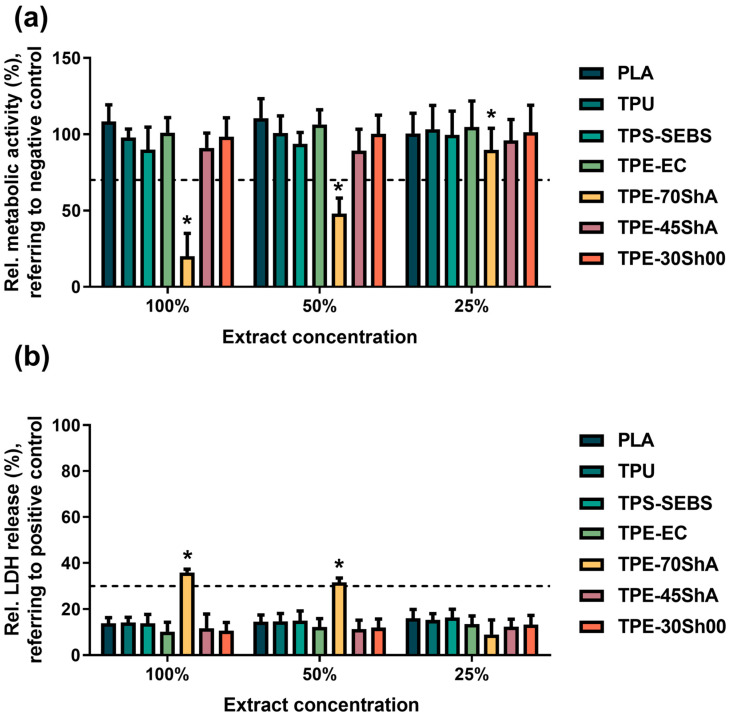
Quantification assessment of extract test. (**a**) Relative cell metabolic activity of L929 cells cultured in different extracts for 24 h, evaluated using a CCK-8 assay. The negative control (Ti) was set to 100%. The dashed line (70% of the negative control) shows a cutoff between toxic and nontoxic effects. * represents a statistical difference compared to the negative control. (**b**) Relative LDH release of L929 cells exposed to different extracts for 24 h, evaluated using an LDH assay. The maximum LDH release was set to 100%. The dashed line (30% of the positive control) indicates a cutoff between toxic and nontoxic effects. * represents a statistical difference compared to the positive control.

Figure 5a shows the relative metabolic activity of L929 cells exposed to the extracts of 3D-printed specimens for 24 h. The one-way ANOVA showed statistically significant differences in relative metabolic activities among the different concentrations (*p* < 0.05). Tukey’s multiple comparisons tests demonstrated that the different diluted extracts (100%, 50%, and 25%) of the TPE-70ShA specimens had significantly lower values compared to the negative control. Also, no significant differences in metabolic activity were found among the other groups, meaning that the values were higher than 70% of the negative control group. In Figure 5b, the relative LDH release was determined, and a similar result was observed. Except for the TPE-70ShA group, the relative LDH release of all the groups was less than 30% that of the negative control. Therefore, the results showed that the sample extracts did not adversely affect cytocompatibility, except for the TPE-70ShA group.

**Figure 6:**
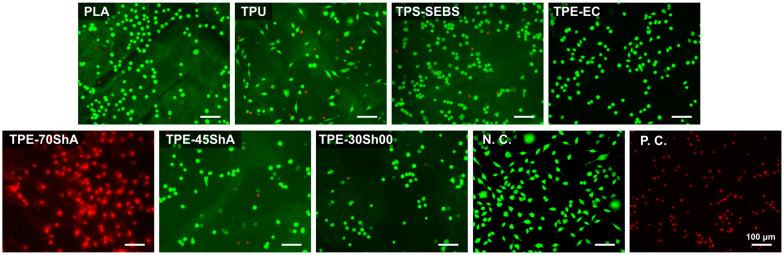
Representative fluorescent staining images of cells cultured on specimen surfaces for 24 h (scale bar = 100 μm). Ti and Cu were the negative control (N.C.) and positive control (P.C.), respectively. Green fluorescence represents viable cells, and red fluorescence indicates apoptotic cells with compromised membrane integrity.

The cell viability of L929 fibroblasts on the surfaces was analyzed using live/dead staining, as depicted in Figure 6. Except for the group of TPE-70ShA, L929 cells with a round or spindle-shaped appearance were attached randomly on the surfaces, in comparison to the negative controls, implying that they were viable cells with membrane integrity. On the contrary, the images of the TPE-70ShA groups showed that most fibroblasts on the surfaces had red fluorescence, similar to the positive control group.

**Figure 7:**
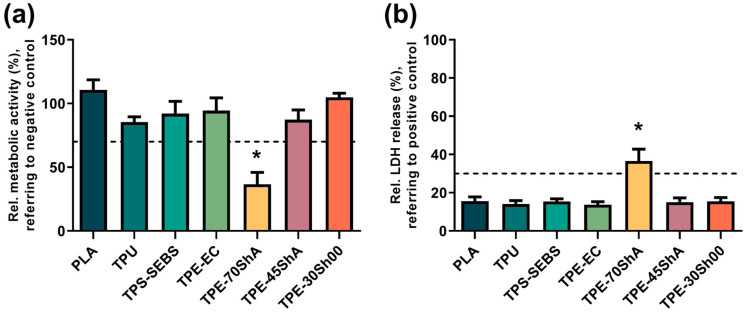
Quantification assessment of direct contact test. (**a**) Relative cell metabolic activity of L929 fibroblasts cultured on specimen surfaces for 24 h, evaluated using a CCK-8 assay. The negative control (Ti) was set to 100%. The dashed line (70% of the negative control) shows a cutoff between toxic and nontoxic effects. * represents a statistical difference compared to the negative control. (**b**) Relative LDH release of L929 fibroblasts cultured on specimen surfaces for 24 h, evaluated using an LDH assay. The maximum LDH release was set to 100%. The dashed line (30% of the positive control) indicates a cutoff between toxic and nontoxic effects. * represents a statistical difference compared to the positive control.

As shown in Figure 7a, the relative metabolic activity of fibroblasts on the surfaces was quantitatively analyzed using a CCK-8 assay. Except for the TPE-70ShA group, no apparent inhibition of the metabolic activities of the cells (>70% of the negative control) was found on the surfaces of 3D-printed specimens. Furthermore, Tukey’s multiple comparisons test showed that the TPE-70ShA group had a significant decrease compared to the negative control (*p* < 0.05). As illustrated in Figure 7b, the results of the LDH release test also confirmed the results, showing that the TPE-70ShA group had significantly higher LDH release values (*p* < 0.05). Therefore, the overall results showed that the 3D-printed specimens exerted no apparent toxic effects on the L929 fibroblasts, except for the TPE-70ShA group.

## 4. Discussion

Technological advancements in additive manufacturing, especially the versatile pellet extrusion method for skin-contact applications, promise to further boost this market. However, the biocompatibility of post-PEX extruded materials warrants further investigation. During the research and development of materials for skin-contact applications, conducting biocompatibility assessments is a critical step to ensure safety. Among these assessments, cytotoxicity testing is particularly crucial.

This study examined the surface properties and cytotoxic effects of seven 3D-printed commercial polymers that varied in hardness, from very soft (TPE-30Sh00) to stiff (PLA). These polymers were evaluated for skin-contact applications using L929 cells. These cells are a standard fibroblast cell line derived from mouse tissue, which is widely utilized in cytotoxicity evaluations, as dictated by ISO 10993-5. The choice of L929 cells was attributed to their consistency and sensitivity. These cells offer a representative response to various chemical substances, allowing for an effective assessment of whether a material releases substances that are harmful to cells. By observing the impact of a given material on L929 cells, it is possible to accurately determine if the material could potentially cause harm to human skin cells.

In our study, several types of TPEs were used. Generally, TPEs are a class of polymers that combine the mechanical properties of rubber with the processing capabilities of thermoplastics. In recent years, their application has expanded into the biomedical field, including uses in medical devices, implants, and skin-contact applications. However, the specific formulation of TPEs, such as the addition of plasticizers, stabilizers, or other additives, can significantly influence their cytotoxicity profiles. In our study, the extract and direct contact tests showed that the TPE-70ShA specimens exhibited clear toxic effects on fibroblasts, which demonstrates the significant toxicity of this material. Compared to other tested materials, the composition of the TPE-70ShA specimen could induce these toxic effects. Moreover, unlike the other materials tested, TPE-70ShA and TPE-EC contain a black colorant. For TPE-EC, the reason for the black color is the carbon black used to promote electrical conductivity. On the other hand, the coloring agent used in TPE-70ShA could play a role in affecting biocompatibility. According to the safety data sheet, despite the absence of hazardous ingredients in TPE-70ShA, the cytotoxic effects observed in our experiments may be attributed to the additives present in TPE-70ShA. Admittedly, a detailed chemical analysis is required to determine the specific contribution of each component to the observed cytotoxic response.

Surface characteristics of materials can directly determine material properties, such as mechanical strength, solubility, and even cytocompatibilty. The surface characteristics of materials printed with the versatile pellet extrusion method are primarily influenced by the printing parameters, material properties, nozzle size, cooling rates, and the choice of post-processing techniques. Optimizing these factors is critical for achieving the desired surface quality and balancing smoothness and detail in the final printed objects. In this study, poor bonding of the layers during the extrusion-based process had a very strong effect on the surface texture of the additively manufactured components. This, in turn, has an effect on the surface that comes into contact with the patient’s skin. It can be assumed that irregular or open surfaces generate different friction on the skin [25,26]. This, in turn, can influence patient comfort. Furthermore, it can also be assumed that open surfaces can form a better hold or niche for biofilms [27]. More biofilm on the surfaces also has a strong influence on hygiene and the associated patient satisfaction. Furthermore, this can lead to skin irritation or other skin diseases. Good cleanability and possible antibacterial surface properties [28] would be a major advantage for 3D-printed components.

One-way ANOVA revealed statistically significant differences in Sa values among specimens (*p* < 0.0001). Additionally, the TPE-70ShA and PLA groups exhibited the lowest Sa values of 4.93 µm and 6.28 µm, respectively.

Admittedly, this study has its limitations, as commercial materials were utilized for the tests, leaving the specific effects of the individual ingredients on cytocompatibility unknown. Moreover, future research should explore additional roughness parameters, such as void volume, which could affect water sorption [29]. While the cytocompatibility of materials intended for skin contact is essential, further investigation into sensitization and irritation/intracutaneous reactivity tests, in accordance with ISO standards, is also necessary.

Notably, the versatile pellet extrusion method, as a technique for 3D printing, primarily aims to provide a versatile and efficient way to fabricate objects. In this study, the commercial polymers, i.e., PLA, TPU, and TPE, were used in the pellet extrusion method. The relative metabolic activity of fibroblasts on the surfaces was quantitatively analyzed using a CCK-8 assay. Except for the TPE-70ShA group, no significant inhibition of cell metabolic activity was observed, with all groups maintaining >70% metabolic activity compared to the negative control. Tukey’s multiple comparisons test indicated that the TPE-70ShA group exhibited a significant decrease in metabolic activity compared to the negative control (*p* < 0.05). Additionally, as illustrated in Figure 7b, the LDH release results confirmed these findings, showing significantly higher LDH values for the TPE-70ShA group (*p* < 0.05). Therefore, the overall results demonstrate that the 3D-printed specimens exerted no apparent toxic effects on L929 fibroblasts, with the exception of the TPE-70ShA group. Furthermore, alterations in surface morphology, potential reductions in residual stresses, or the introduction of specific surface characteristics, as facilitated by additive manufacturing techniques, do not inherently cause cytotoxicity. Instead, these factors may enhance cell attachment and proliferation, promoting biocompatibility. However, careful consideration of material composition, processing conditions, and post-processing techniques remains crucial in ensuring the safety and efficacy of 3D-printed materials for biomedical applications, particularly those intended for skin-contact applications. Further research on the specific mechanisms underlying cytotoxicity, as well as the optimization of additive manufacturing parameters, will contribute to the continued advancement of biocompatible 3D-printed materials.

## Figures and Tables

**Table 1:** Materials in the form of pellets used for the biocompatibility test.

No	Material	Material Trade Name	Company	Hardness	Abbreviation
#1	Polylactide natural	PLA Nat	Pollen AM	~70 ShoreD	PLA
#2	Thermoplastic polyurethane	TPU High Strength	Pollen AM	85 ShoreA	TPU
#3	Thermoplastic styrene block copolymers	Green flex 608353-2	HEXPOL	60 ShoreA	TPS-SEBS
#4	Thermoplastic elastomer with carbon black content	TC7OEX-BLCK (EC Series)	Kraiburg TPE	70 ShoreA	TPE-EC
#5	Thermoplastic elastomer	TC7FTZ (FR2 Series)	Kraiburg TPE	70 ShoreA	TPE-70ShA
#6	Thermoplastic elastomer	TPE 45 ShA	Pollen AM	45 ShoreA	TPE-45ShA
#7	Thermoplastic elastomer	TPE 30 Sh00	Pollen AM	30 Shore00	TPE-30Sh00

**Table 2:** Printing parameters. The 3D printer has variable ± 10 °C for all the temperatures.

Material	PLA	TPU	TPS-SEBS	TPE-EC	TPE-70ShA	TPE-45ShA	TPE-30Sh00
Cold temperature [°C]	62	65	50	57	45	57	45
Extruder temperature [°C]	167	178	110	130	110	130	130
Head temperature [°C]	185	210	200	225	180	220	195
Bed temperature [°C]	60	60	70	60	60	60	35
Flow [%]	50	55	48	270	43	53	45
Printing speed [mm/s]	20	25	20	15	20	15	15

## Data Availability

The data presented in this study are available on request from the corresponding author.
